# Awareness of the Warning Signs, Risk Factors, and Treatment for Tuberculosis among Urban Nigerians

**DOI:** 10.1155/2013/369717

**Published:** 2013-01-14

**Authors:** Olufemi O. Desalu, Adekunle O. Adeoti, Abayomi Fadeyi, Alakija K. Salami, Ademola E. Fawibe, Olanrewaju O. Oyedepo

**Affiliations:** ^1^Department of Medicine, University of Ilorin Teaching Hospital, PMB 1459, Ilorin 240001, Nigeria; ^2^Department of Medicine, Ekiti State University Teaching Hospital, PMB 5355, Ado-Ekiti, Nigeria; ^3^Department of Medical Microbiology, University of Ilorin Teaching Hospital, PMB 1459, Ilorin 240001, Nigeria; ^4^Department of Anaesthesia, University of Ilorin Teaching Hospital, PMB 1459, Ilorin 240001, Nigeria

## Abstract

*Objectives*. To determine the awareness of the warning signs, risk factors, and treatment of tuberculosis among urban Nigerians. *Methods*. This was a cross-sectional survey among 574 adults in Ilorin, Nigeria. Semistructured questionnaire was administered by trained interviewers to obtain information about awareness of tuberculosis warning signs, risk factors, and treatment. *Results*. Majority of the subjects (71.4%) were aware of at least one warning sign of tuberculosis. Cough (66.2%), weight loss (38.0%), and haemoptysis (30.7%) were the most identified warning signs. The predictors of awareness of warning sign were increasing age (*r* + 0.12), higher family income (*r* + 0.10), higher level of education (*r* + 0.10), and belonging to Christian faith (*r* + 0.11). Awareness of risk factors for tuberculosis was higher for tobacco smokers (77.0%) and history of contact with a case of TB (76.0%). Less than half were aware of HIV infection (49.8%), alcohol consumption (42.5%), chronic kidney disease (40.4%), extremes of ages (39.4%), cancers (36.9%), and diabetes mellitus (27.5%) as risk factors for TB. Tuberculosis was reported to be curable by 74.6% of the subjects and 67.9% knew that there are medications for treatment of tuberculosis, while 11.5% knew the duration of treatment. *Conclusion*. This study has revealed that the awareness of HIV and noncommunicable diseases as risk factors for TB is poor. This study has therefore demonstrated the need for health education programs that will emphasize recognition, identification, and modification of risk factor for TB.

## 1. Introduction

Tuberculosis (TB) is a chronic infectious disease caused by a bacterium called *Mycobacterium tuberculosis*. It usually affects the lungs in 80% of cases with warning signs of cough, haemoptysis, and chest pain, shortness of breath, fever, weight loss, and drenching night sweat [1]. TB is spread mainly through the air inform of droplets. When infectious people cough, sneeze, talk, laugh or spit, droplets containing *Mycobacterium tuberculosis* are sprayed into the air. People nearby may inhale the bacteria and become infected. *Mycobacterium tuberculosis* can remain viable as air-borne droplet suspended in the air for a long time or as part of house dust for weeks. However, transmission usually occurs only after substantial exposure to someone with active TB [1, 2]. A person can be infected by *Mycobacterium tuberculosis* for many years without getting sick or spreading the organism to other people. If the immune system is weakened by immunosuppressive disease like HIV infection, diabetes mellitus, malignancy, chronic kidney disease, extremes of ages, and immunosuppressive agent, latent TB infection can develop into active disease [1, 2]. If a person with active disease is left untreated, he or she will infect on the average between 10 and 15 people every year [3]. TB accounts for 2.5% of the global burden of disease and is the commonest cause of death in young women, killing more women than all causes of maternal mortality combined [4–6]. Ninety-five per cent of all cases and 99% of deaths occur in developing countries, with the greatest burden in sub-Saharan Africa and South East Asia [7]. It currently holds the seventh place in the global ranking of causes of death. Unless intensive efforts are made, it is likely to maintain that position through to 2020, despite a substantial projected decline in disease burden from other infectious diseases [5, 6]. TB causes a significant socioeconomic burden: as three quarter of cases are within the economically productive age group of 15–54 years [8]. Tuberculosis is a serious public health problem in Nigeria with an estimated prevalence of nearly 900,000 TB cases and with the second highest TB disease burden in Africa and ranks 5th among the 22 high TB burden countries in the world [4]. In Nigeria, there were 90,447 TB cases notified in 2010 with 41, 416 (58%) cases as new smear positives, and a case detection rate of 40%. Ilorin city is the capital of Kwara State, a state that has one of the least TB notification rate (900 per annum) when compared with other states in the country like Lagos (8200 per annum) and Oyo (6000 per annum) that have the higher TB notification rate [9]. The outcome of TB control is determined by the clinical and social factors like delayed presentation and health care utilization which depend on the knowledge and awareness of TB symptoms among the population [10, 11]. For us to meet the Millennium Development Goal (MDG), the Global Plan to Stop TB and reverse the incidence of TB by 2015, we need to determine the level of awareness of the warning signs, risk factors, and treatment of tuberculosis among the population as modifiable factors in the TB control program. Therefore the objectives of our study were to determine the awareness of the warning signs, risk factors, and treatment of tuberculosis among urban residents in Ilorin, Nigeria.

## 2. Materials and Methods

This was a cross-sectional survey among subjects aged ≥18 years who reside in Ilorin West and East local government area Kwara state, Nigeria that was conducted between July 2011 and August 2011. The subjects were recruited by multistage stratified sampling method. A minimum sample size of 384 was arrived at using the Cochran formula *N* = *Z*2*pq*/*d*2, where *N* was the required sample size; *p* was (50%) [5]; *q*, (1–*p*); *Z*, an SD of 1.96 (which corresponds to a 95% confidence interval); and *d* is the degree of accuracy desired (0.05 for an acceptable error margin of 5%). The design effect of 1.0 which was derived from the homogeneity of the two council areas was used to adjust the minimum sample size. The local government areas consist of electoral wards that were divided into clusters and a sample frame containing the list of clusters was constructed by the investigators. The households in each cluster were randomly selected. The adults in each household were informed about the study by our trained interviewers and a maximum of two persons per household were selected in order to have normal spread and true representation of the study population. We then recruited individuals who met the inclusion criteria (age of 18–45 years, resided in Ilorin for at least 1 year, and willingness to participate in the study) from the selected households. Those who participated in the pretesting of the questionnaire and those with a past history of TB were excluded from the study as it was assumed that the former group would have been primed to seek for information on warning signs and risk factors for TB while the latter category would have a better knowledge of the warning signs having previously suffered from the disease. 

A pretested semistructured questionnaire was administered face to face by trained interviewers in their place of residence or work. The questionnaire was in English, which is the official language of communication in Nigeria and translated to Yoruba language for few respondents who could not communicate in English. The questionnaire was used to obtain sociodemographic information, awareness of the warning sign of tuberculosis, the predisposing risk factors, and TB medication, cure and treatment duration. The question on awareness of the disease was based mostly on nonpersonal experience (individual level of enlightenment and public health education). 

## 3. Data Analysis

The data were analyzed using SPSS version 15, Chicago Inc., IL, USA. Frequency statistics was performed for sociodemographic variables warning sign and risk factor for TB in the study population. The warning symptoms were analyzed according to gender using 2 × 2 cross tab, and chi square was calculated to determine the level of significance. *P* < 0.05 was considered to be statistically significant. Spearman correlation coefficient was used to determine the predictors of warning sign of TB. The confounding variables were identified by stratification of variables and they were used in adjusting the spearman correlation coefficient.

## 4. Results 

A total of 574 subjects participated in the study; their median age was 30 with an interquartile range of (23–40). Of the 574, 308 (53.7%) were males and majority were unskilled workers and they earned below fifty thousand naira per month (three hundred and twelve dollars per month). 

Majorities (459; 80%) were aware of TB as a disease. Four hundred and ten (71.4%) subjects were aware of at least one TB warning sign (Table 1). Of the 410 that were aware of the TB warning sign, 228 (55.6%) were males and 182 (44.4) were females. Majority of the subjects (66.2%) were aware of cough as TB warning sign, 218 (38.0%) were aware of weight loss, and 176 (30.7%) were aware of haemoptysis. Chronic cough was commonly reported as a warning sign by men (56; 18.2%) compared with women (18; 11.3%). Similarly, fever was commonly recognized by men (40; 13.0% versus 14; 5.3%) compared with women (Table 2). 

The predictors of awareness of TB warning sign were increasing age (*r* + 0.12), higher family income (*r* + 0.10), higher level of education (*r* + 0.10), and belonging to Christian faith (*r* + 0.11) (Table 3).

In this study, 442 (77.0%) subjects reported active tobacco smoking as a risk factor for TB, 436 (76.0%) reported history of contact with an index case of TB or chronic cough, while 342 (59.6%) cited health care related occupation as a risk factor for TB. Three hundred and forty-four (59.0%) were aware of overcrowding and likewise environmental tobacco smoking as risk factor for TB, respectively. The use of biomass fuel as source of cooking energy was also reported by 326 (56.8%). Less than half of the subjects were aware of HIV infection (49.8%), alcohol consumption (42.5%), CKD/CRF (40.4%), extremes of ages (39.4%), and cancer (36.9%) as risk factors for TB. One hundred and fifty-eight subjects (27.5%) were aware of diabetes mellitus as risk factor for TB (Table 4).

In the treatment of TB, 428 (74.6%) reported that TB is curable, 390 (67.9%) were aware that there are drugs for treatment of tuberculosis, and only 66 (11.5%) knew the duration of treatment. 

## 5. Discussion

This present study was undertaken to determine the public awareness of warning symptoms, risk factors, and treatment of TB in Ilorin, Nigeria. Few studies on the awareness of TB exist in north-central Nigeria [12]. We defined TB warning sign as something perceived that informs or serves to warn a person about the presence or a future occurrence of tuberculosis.

In this study, about 71% of the subjects were aware of at least one warning sign of tuberculosis and about 29% could not mention a warning sign of TB. This result of this study is closer to the one by Vukovic et al. [13], among 1129 adults in Serbia where they found that almost a third of the subjects could not identify any sign of TB. Further analysis by gender showed that more men were aware of TB warning sign than the women. This pattern of observation may be explained by the result of Nigerian demographic health survey of 2008 which noted that in the same study region about 81% of men have heard of TB compared with 52% of women [12]. 

We also found that the most commonly identified TB warning sign was cough followed by weight loss and haemoptysis. Similar studies in Asia have also noted cough and haemoptysis as the common warning signs or symptoms of tuberculosis [14–18]. The most common warning sign in this study is cough and it is in contrast to what was reported in the Malaysian study where the respondents noted haemoptysis as the most common sign of TB [14]. Geographical variation in the awareness of TB warning sign may be attributed to the discrepancy in the burden of the disease in both these two countries, prior knowledge of TB, personal experience with a close friend, and family members affected by the disease. It also depends on sources of public health information. 

The significant predictors of awareness of TB warning sign were increasing age, being a Christian, higher family income, and education. The association of increasing age with awareness of TB sign may be attributed to increasing knowledge of TB and lifetime experiences of close relative who had the disease. We also discovered that those who belong to Christian faith were more likely to be aware of TB sign than others after adjusting for confounding variable like age, sex, income, and level of education. Further subanalysis by stratification of the religion, was suffice to explain the religious predisposition as 0.8% of the Christians were uneducated compared with the Muslims that had 20.8% uneducated subjects in this study.

The association of higher family income and education with awareness of TB signs may be attributed to higher literacy. Higher level of educational attainment is often a factor for better family income. Families that have high income are able to purchase household assets like television, radio and Hi fi internet, and other communication appliances that increase their knowledge of health-related matters which are of public concern. Similar studies have also supported our observation that literates were more likely to be aware than the illiterates of signs and symptoms of TB [13, 16, 17, 19, 20]. A recent study in Pakistan also noted the association of higher monthly income with the knowledge of TB [21]. 

Our result also revealed that more than half of the subjects were aware of tobacco smoking (active and secondhand), history of contact, health care job, household overcrowding, use of biomass fuel as a source of cooking energy, work-related pollution, and low family income as a risk factor for TB. More than 50% were unaware of noncommunicable diseases (NCDs) like diabetes mellitus, cancers, chronic kidney disease, and behavioural factors like alcohol as risk factors for TB. Surprisingly half of the subjects did not report HIV which has a higher burden in sub-Saharan Africa as risk factor for TB. The NCDs are expected to increase by 2030 and as the impact of NCDs increases, and as population's age, annual NCD deaths are projected to continue to rise worldwide, and the greatest increase is expected to be seen in low- and middle-income regions. An extreme age of life was also cited by less than half of the subjects [22, 23]. Studies in Morocco [24] and Pakistan [17] have reported that significant percentages of the respondents in their study were unable to identify this as risk factor for TB. With regards to tuberculosis therapy, approximately 75% of the subjects reported that TB is curable, 67.9% were aware that there are drugs for treatment of tuberculosis, and only 11.5% knew the 6–8 months duration of treatment. Respondents in several studies have also noted TB as a curable disease [13, 21, 24, 25]. Singh et al. in 2002 [26] also reported that only 12.6% of their subjects in India knew the duration of TB therapy which is in agreement with 11.6% obtained in our study.

This study has determined the awareness of the warning sign, risk factor, and treatment for tuberculosis; however we have not explored the sociocultural belief and misconception of tuberculosis in the era of directly observed therapy which is not part of the objective of this study. This might be important direction in terms of research. 

The study is limited by the small size of the population and power of the study, and nonvalidation of the study questionnaire in local language. The extent to which the translated items in questionnaire measure what it was intended to measure has not been statistically validated. 

## 6. Conclusion

This study has shown the high level of awareness of TB warning sign and TB as a curable disease among the studied population. HIV, non communicable diseases like DM, alcohol consumption, and cancers were poorly reported as risk factors for TB.

## Figures and Tables

**Table 1:** General characteristics of the study subjects.

Characteristics	*n* (%)
Age	30 (23–40)*
Sex	
Male	308 (53.7)
Female	266 (46.6)
Education	
Primary/none	150 (26.1)
Secondary	148 (25.8)
Tertiary	276 (48.1)
Income (*N*)**	
<10,000	244 (42.5)
≤50,000	206 (39.5)
≤100,000	72 (12.5)
≤200,000	26 (4.5)
>*N*200,000	22 (3.8)
Occupational class	
Professional/administrators	21 (3.7)
Skilled	32 (5.6)
Unskilled	100 (17.4)
Others	421 (73.3)
Marital status	
Married	252 (43.9)
Others	322 (56.1)
Religion	
Islam	336 (58.5)
Christianity	236 (41.1)
Others	2 (0.3)
Aware of TB as a disease	
Aware	459 (80.0)
Unaware	115 (20.0)
Awareness of TB sign	
Aware	410 (71.4)
Unaware	164 (28.6)

*Median (interquartile range).

**4 missing.

**Table 2:** Identified TB warning sign according to gender.

Warning sign	Male (%) *N* = 308	Female (%) *N* = 266	Total (%) *N* = 574
Cough	212 (68.8)	168 (63.2)	380 (66.2)
Weight loss	126 (40.9)	92 (34.6)	218 (38.0)
Heamoptysis	104 (33.8)	72 (27.1)	176 (30.7)
Chronic cough	56 (18.2)	30 (11.3)	86 (15.0)*
Breathlessness	44 (14.3)	34 (12.8)	78 (13.6)
Chest pain	32 (10.4)	28 (10.5)	60 (10.5)
Fever	40 (13.0)	14 (5.3)	54 (9.4)*
Fatigue	16 (5.2)	20 (7.5)	36 (6.3)
Night sweat	20 (6.5)	10 (3.8)	30 (5.2)

*P < 0.05.

**Table 3:** Predictors of warning signs of TB.

Predictors	Crude *r* ^2^	Adjusted *r* ^2^
Age	+0.13*	+0.12*
Sex	+0.07	+0.06
Higher level of education	+0.06	+0.10*
Unskilled workers	+0.02	+0.03
Marriage	+0.04	+0.04
Higher family income	+0.18*	+0.10*
Christianity	+0.20*	+0.11*
Having a medical disease	+0.01	−0.04

Adjusted for age, sex, religion, and sociodemographic variables.

*P < 0.05.

**Table 4:** Awareness of the risk factors for TB in the subjects.

Reported risk factor	*n* (%)
Tobacco smoking	
-Active	442 (77.0)
-ETS	344 (59.0)
History of contact	436 (76.0)
Health care job	342 (59.6)
Overcrowding	344 (59.0)
Use of biomass fuel	326 (56.8)
Work-related pollution	322 (56.1)
Low income	312 (54.4)
HIV infection	286 (49.8)
Alcohol intake	244 (42.5)
CKD/CRF	232 (40.4)
Extremes ages	226 (39.4)
Cancer	212 (36.9)
Diabetes mellitus	158 (27.5)
